# Aging- and injury-related differential apoptotic response in the dentate gyrus of the hippocampus in rats following brain trauma

**DOI:** 10.3389/fnagi.2013.00095

**Published:** 2013-12-18

**Authors:** Dong Sun, Melissa McGinn, Jeanette E. Hankins, Katherine M. Mays, Andrew Rolfe, Raymond J. Colello

**Affiliations:** ^1^Department of Neurosurgery, Medical College of Virginia Campus, Virginia Commonwealth UniversityRichmond, VA, USA; ^2^Departments of Anatomy and Neurobiology, Medical College of Virginia Campus, Virginia Commonwealth UniversityRichmond, VA, USA

**Keywords:** aging, apoptosis, dentate gyrus, neurogenesis, proteomics, traumatic brain injury

## Abstract

The elderly are among the most vulnerable to traumatic brain injury (TBI) with poor functional outcomes and impaired cognitive recovery. Of the pathological changes that occur following TBI, apoptosis is an important contributor to the secondary insults and subsequent morbidity associated with TBI. The current study investigated age-related differences in the apoptotic response to injury, which may represent a mechanistic underpinning of the heightened vulnerability of the aged brain to TBI. This study compared the degree of TBI-induced apoptotic response and changes of several apoptosis-related proteins in the hippocampal dentate gyrus (DG) of juvenile and aged animals following injury. Juvenile (p28) and aged rats (24 months) were subjected to a moderate fluid percussive injury or sham injury and sacrificed at 2 days post-injury. One group of rats in both ages was sacrificed and brain sections were processed for TUNEL and immunofluorescent labeling to assess the level of apoptosis and to identify cell types which undergo apoptosis. Another group of animals was subjected to proteomic analysis, whereby proteins from the ipsilateral DG were extracted and subjected to 2D-gel electrophoresis and mass spectrometry analysis. Histological studies revealed age- and injury-related differences in the number of TUNEL-labeled cells in the DG. In sham animals, juveniles displayed a higher number of TUNEL^+^ apoptotic cells located primarily in the subgranular zone of the DG as compared to the aged brain. These apoptotic cells expressed the early neuronal marker PSA-NCAM, suggestive of newly generated immature neurons. In contrast, aged rats had a significantly higher number of TUNEL^+^ cells following TBI than injured juveniles, which were NeuN-positive mature neurons located predominantly in the granule cell layer. Fluorescent triple labeling revealed that microglial cells were closely associated to the apoptotic cells. In concert with these cellular changes, proteomic studies revealed both age-associated and injury-induced changes in the expression levels of three apoptotic-related proteins: hippocalcin, leucine-rich acidic nuclear protein and heat shock protein 27. Taken together, this study revealed distinct apoptotic responses following TBI in the juvenile and aged brain which may contribute to the differential cognitive recovery observed.

## INTRODUCTION

Traumatic brain injury (TBI) is a major cause of death and disability world-wide. With the increase in numbers of the aging population in the United States, the epidemiology of TBI has shifted toward this demographic, with falls representing the leading cause of brain injuries involving the elderly. Although no overt neuronal cell loss is observed in the brain with aging, several subtle structural, chemical, and metabolic changes occur that render the aged brain more vulnerable to TBI as compared to the young brain. These changes include a reduction in the complexity of dendritic arborization, dendritic length, and spine numbers (Dickstein et al., 2007), increased oxidative stress and altered metabolic functions (Mattson and Magnus, 2006), and increased glial cell reaction and neuroinflammation (Frank et al., 2006). Collectively, these changes result in less plasticity and repair potential for the aged following TBI and lead to more enduring functional deficits.

The hippocampus, a region responsible for learning and memory functions, is particularly vulnerable to TBI. The learning and memory deficits observed following TBI are likely a reflection of differential susceptibility of neurons in different hippocampal subregions to injury (Small et al., 2011). Under normal conditions, new cells are constantly generated in the dentate gyrus (DG) in the hippocampus. Of these newly generated granule cells, approximately half of them die via apoptosis within the first month following their generation (Dayer et al., 2003); those that survive ultimately mature into functional granule neurons (van Pragg et al., 2002; Ramirez-Amaya et al., 2006) involved in hippocampal-dependent learning and memory functions (Clelland et al., 2009; Deng et al., 2009). With normal aging, the neurogenic capacity of the DG is significantly decreased, with a concomitant increased vulnerability of neurons in this region and the decline in cognitive function (Pavlopoulos et al., 2013). Following TBI, in young adult animals, the injured brain displays a significantly enhanced neurogenic response in the DG (Chirumamilla et al., 2002; Sun et al., 2005). However, heightened levels of hippocampal neuronal degeneration and cell death, particularly among the newly generated neurons in the DG, are also observed following TBI (Gao et al., 2008).

As neuronal generation and degeneration concomitantly exist following TBI, the observed age-related differences in recovery may be due to not only the level of neurogenesis but also differences in the degree of cell death occurring after brain injury. Neural cell loss in the hippocampus has been linked to multiple neurochemical pathways and cell death cascades leading to necrosis and apoptosis (Raghupathi, 2004). Apart from necrotic cell death due to focal tissue damage following TBI, cell death consistent with apoptosis has been observed in the cortex, hippocampus, and thalamus both in clinical and experimental brain injury (Clark et al., 1997; Conti et al., 1998; Fox et al., 1998; Newcomb et al., 1999). Underscoring the incredible scope of the cellular response after injury is the finding that apoptotic neurons have been observed in the human hippocampus up to 12 months after injury (Williams et al., 2001).

In order to ascertain the potential link between apoptotic cell death and the observed age-related differences in functional recovery following TBI, the current study was undertaken to investigate the levels of aging and injury associated apoptotic cell death and the proteomic profiles of apoptosis-related proteins in the DG of the juvenile and aged rats.

## MATERIALS AND METHODS

### ANIMALS

Juvenile (postnatal day 28; weighing approximately 70 g) and aged (24 months; weighing approximately 575 g) male Sprague–Dawley rats (Harlan Inc., IN, USA) were used. Animals were housed in the animal facility, with a 12-h light/dark cycle, water and food provided *ad libitum*. All procedures were approved by our Institutional Animal Care and Use Committee.

### FLUID PERCUSSION INJURY

Animals were subjected to moderate TBI (*n* = 7 for each age group) or sham injury (*n* = 7 for each age group) using the lateral fluid percussion injury (FPI) model as previously described (Chirumamilla et al., 2002; Sun et al., 2005, 2007). Briefly, rats were anesthetized in a plexiglass chamber with 3% isofluorane in 30% O_2_/70% N_2_, intubated and ventilated with 2% isofluorane in 30% O_2_/70% N_2_ and secured in a stereotaxic frame. Since intubation was not feasible in juvenile rats, these animals received continuous anesthesia via nose cone with the gas mixture described above. A midline incision was made to expose the skull and a 4.9 mm craniotomy was made on the left parietal bone halfway between the sutural landmarks lambda and bregma. A modified Luer lock fitting was then secured to the skull using cyanoacrylate adhesive and dental acrylic. A moderate fluid pressure pulse (2.00 ± 0.05 Atm) was administered through the craniotomy onto the intact dura, using a pre-calibrated FPI device. After injury, the Luer lock fitting was removed, the wound sutured and after a 3-h observation, the rats were returned to the vivarium. Sham animals underwent the same surgical procedure, but did not receive the injury pulse. Animals were allowed to survive for 48 h following injury, at which point they were anesthetized and brain tissue processed for either histochemical or proteomic analysis.

### TISSUE PREPARATION FOR HISTOCHEMICAL PROCEDURES

Forty-eight hours following injury, animals (*n* = 4/group) were anesthetized with isofluorane, euthanized with euthasol (pentobarbital sodium, 780 mg/kg; phenytoin sodium 100 mg/kg), and transcardially perfused with phosphate buffered saline (PBS) immediately followed by 1% paraformaldehyde in PBS. The brains were rapidly dissected and immediately frozen in dry ice-chilled isopentane at -30°C. Ten μm-thick coronal sections spanning the rostro-caudal extent of the hippocampal DG (corresponding to the Paxinos and Watson stereotaxic rat atlas coordinates of -2.5 to -5.2 relative to bregma (Paxinos and Watson, 1986) were cut by cryostat, collected onto Superfrost^®^/Plus Slides (Fisher Scientific) and stored at -80°C until histochemical procedures were conducted.

### TUNEL HISTOCHEMISTRY

In order to assess apoptosis levels in the DG, TUNEL histochemistry was performed according to the manufacturer’s protocol using the ApopTag^®^ Plus Fluorescein *In Situ* Apoptosis Detection Kit (Millipore, Billerica, MA, USA). Briefly, sections were post-fixed in pre-cooled ethanol:acetic acid (2:1) for 5 mins at -20°C, followed by two 5 mins washes in PBS. An equilibration buffer was applied for 10 s followed by 1 h incubation in a labeling solution of working strength TdT enzyme (composed of reaction buffer and TdT enzyme) in a humidified 37°C chamber. Following a 15 s agitation, sections were incubated for 10 mins in the stop/wash buffer to terminate the reaction and subsequently washed three times (one minute each) in PBS. In order to visualize the DNA fragments, sections were incubated in working strength anti-digoxigenin conjugate (composed of blocking solution and anti-digoxigenin antibody conjugated to fluorescein) for 30 min in a dark, humidified chamber at room temperature. Following four PBS washes, a mounting medium containing 0.5 μg/mL of a DAPI nuclear counter stain (Vector Lab, Burlingame, CA, USA) was applied to sections and slides were coverslipped.

### QUANTIFICATION OF APOPTOTIC CELLS

**FIGURE 1:**
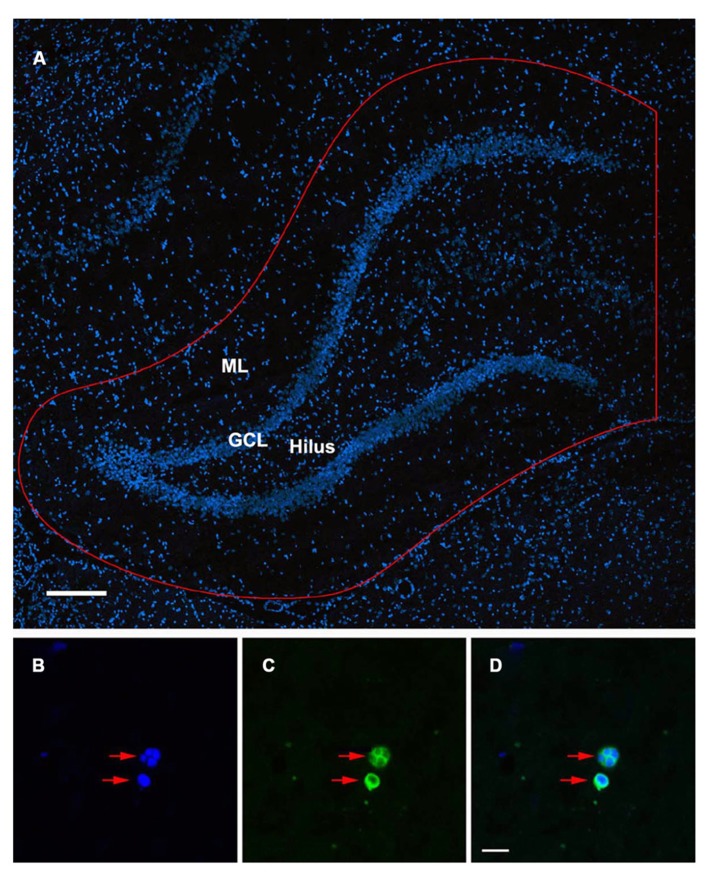
**TUNEL-labeled and DAPI-stained nuclei of the rat dentate gyrus.** **(A) **Confocal image showing DAPI-stained nuclei in the dentate gyrus of the ipsilateral hippocampus. Sampling region of interest for quantification is outlined in red including the molecular layers (ML), suprapyramidal and infrapyramidal blades, granule cell layer (GCL), subgranular zone, and hilus of the dentate gyrus. Scale bar = 200 μm. **(B–D) **Condensed, fragmented nuclei exhibiting the morphological characteristics of apoptosis (red arrows) are stained with DAPI **(B)** and TUNEL **(C)**. The co-localization of DAPI/ TUNEL **(D)**, in combination with the morphological features of the labeled nuclei would indicate an apoptotic phenotype. Scale bar = 10 μm.

TUNEL-stained sections were examined at 40× with an Olympus BX51 microscope with a BX-RFA fluorescence illuminator (Olympus, Tokyo, Japan). To quantify the degree of apoptotic cell death in the DG, every tenth coronal section throughout the rostro-caudal extent of the hippocampus was examined (for a total of 25 sections at 10 μm thickness per brain) and TUNEL-positive cells were systematically counted in a defined sampling region. The sampling region of interest extended from the hippocampal fissure down to the border of the lateral ventricle, including the suprapyramidal and infrapyramidal blades, granule cell layer (GCL), subgranular zone, and hilus of the DG (**Figure 1A**). A cell was considered apoptotic only if its nucleus was labeled with both fluorescein and a DAPI nuclear counter stain. The apoptotic state of these TUNEL-positive cells was further confirmed by the presence of condensed or fragmented nuclei when visualized by DAPI (**Figures 1B–D**). The estimated number of TUNEL-positive cells reported in the results represents the sum of apoptotic cell counts from all quantified sections throughout the DG of an individual rat multiplied by 10 to account for the intervals between quantified sections. The number of TUNEL-positive cells reported was the average of four animals in each group. In addition to evaluating TUNEL-positive cells in the DG of injured juvenile and aged animals, the extent of apoptotic cell death was also examined in age-matched shams.

### IMMUNOFLUORESCENT LABELING

To determine the phenotype of cells undergoing apoptosis in the DG following brain injury, triple immunofluorescent labeling was performed with mature neuronal marker NeuN, immature neuronal marker PSA-NCAM, astrocytic marker GFAP, and microglia cell marker Iba1. Briefly, sections were incubated in a blocking solution (5% normal horse serum and 1% bovine serum albumin in PBS) with 1% Triton for 1 h at room temperature. Sections were then incubated overnight at 4°C with mouse monoclonal NeuN (1:500, Millipore), PSA-NCAM (1:500, Millipore), or rabbit polyclonal GFAP (1:1000, Dako) combined with goat polyclonal Iba1 (1:1000, Wako) diluted in the serum blocking solution. Following over night primary antibody incubation at 4°C, sections were washed in PBS and then incubated for 1 h at room temperature with Alex Fluor 568 anti-mouse IgG (for NeuN), IgA (for PSA-NCAM), or anti-rabbit IgG (for GFAP) combined with Alex Fluor 488 anti-goat IgG (1:200, Molecular Probes) in the serum blocking solution. Following incubation, sections were then washed three times in PBS and incubated with DAPI (1:1000) for 10 min. After a PBS wash, sections were coverslipped with Vectorshied. Sections were examined by confocal microscopy (Leica TCS SP2).

### TISSUE PREPARATION FOR PROTEOMIC ANALYSIS

Forty-eight hours following injury, animals (*n* = 3 for TBI and sham animals in each age group) were anesthetized with isofluorane, euthanized with euthasol (pentobarbital sodium, 780 mg/kg; phenytoin sodium 100 mg/kg) and transcardially perfused with ice-cold PBS. Brains were then rapidly dissected on ice and placed in a rat brain mold with coronal divisions, so that three 1 mm coronal sections encompassing the rostro-caudal extent of the DG were cut using a single edge razor blade. The hippocampus ipsilateral to the site of injury in each slice was visualized using an Olympus SZX9 dissecting microscope and the dentate gyri (corresponding to the sampling region of interest described above for TUNEL analysis) from the three slices were dissected, pooled, and snap frozen on dry ice. This was repeated on three samples for each of the four groups (young sham, aged sham, young TBI, and aged TBI). Tissues samples were then thawed on ice and homogenized in an osmotic lysis buffer containing protease inhibitors and nucleases. Samples were quantified with a bicinchoninic acid (BCA) protein assay to determine the protein concentration of each sample and to ensure that equal quantities of proteins (50 μg) were loaded onto each gel for two dimensional polyacrylamide gel electrophoresis (2D-PAGE).

### PROTEOMIC ANALYSIS

In order to assess group differences in protein expression profiles, a comparative proteomic analysis was performed on tissue extracted from the DG of juvenile and aged rats subjected to either a sham or FPI. To identify candidate proteins in the DG that exhibited differential expression patterns with aging and injury, tissue was processed for 2D-PAGE (by Kendrick Laboratories, Madison, WI, USA) according to the method of O’Farrell (1975) and stained with a mass spectrometry (MS)-compatible special silver stain according to the Vorum method as previously described (Colello et al., 2002). 2D-PAGE, which separates proteins based first according to charge and then according to molecular weight (MW), was performed on 10% acrylamide slab gels capable of resolving proteins in the 15–200 kDa MW range with an isoelectric point (pI) between 3.5 and 10. 50 ng of an internal standard (purified tropomyosin – MW 33,000; pI 5.2) was added to the samples before gel running to serve as a reference marker. Duplicate 2D gel proteomic map sets were generated from DG tissue derived from both sham and fluid percussion injured animals of both age groups (*n* = 3/group) resulting in a total of six gels per experimental group.

The objective of this proteomic analysis was twofold: (1) to identify apoptosis-related proteins that are differentially expressed in the DG during the normal aging process and (2) to identify apoptosis-associated proteins that exhibit age-dependent alterations in expression following TBI. To this end, a manual and computer-automated subtractive comparative analysis was performed using Discovery Series PDQuest 2D-Gel Analysis software (Version 7.3.1, Bio-Rad, Hercules, CA, USA). Briefly, gels were digitized using a Bio-Rad GS-800 scanner (BioRad) and digital gels were cropped, prepped for spot detection, filtered, and smoothed to clarify spots using PDQuest software. Spot volume and density (optical density) parameters were used to quantitatively compare corresponding protein spots between experimental groups. To account for any inconsistencies in silver staining, the total staining intensity in a gel image was used to normalize spot density. Optical density measurements were further normalized via background subtraction and according to the internal tropomyosin standard. A conservative twofold selection threshold was applied to control the number of 2D gel spots processed for tandem mass spectrometric identification. Thus, only the more prominent protein changes were selected in this initial study and were subsequently excised from the 2D gels, tryptic digested, and processed for mass spectrometric analysis.

Liquid chromatography-electrospray ionization-tandem MS (LC-ESI-MS/MS; performed by the Stanford Mass Spectrometry Laboratory, Stanford University) was then used to determine the amino acid sequence of each protein, which was then compared to theoretical MS spectra of known proteins using a MASCOT database search in order to determine the identity of the protein.

### DATA ANALYSIS

The TUNEL data was analyzed using SPSS software with analysis of variance (ANOVA) with *post hoc* Fisher LSD test or the Student *t*-test with an applied Bonferroni correction for multiple groups was utilized, with *p* value less than 0.05 considered statistically significant. Densitometric data for identified gel spots for log(2) transformed to a normal distribution and tested using a two-way ANOVA method for factors of injury and age and the interaction between the two using a Holm–Sidak method for multiple comparisons based on an initial alpha of 0.05. All values are reported as mean ± SEM in all figures.

## RESULTS

### INCREASED NUMBERS OF APOPTOTIC CELLS IN THE AGED BRAIN FOLLOWING TBI

**FIGURE 2:**
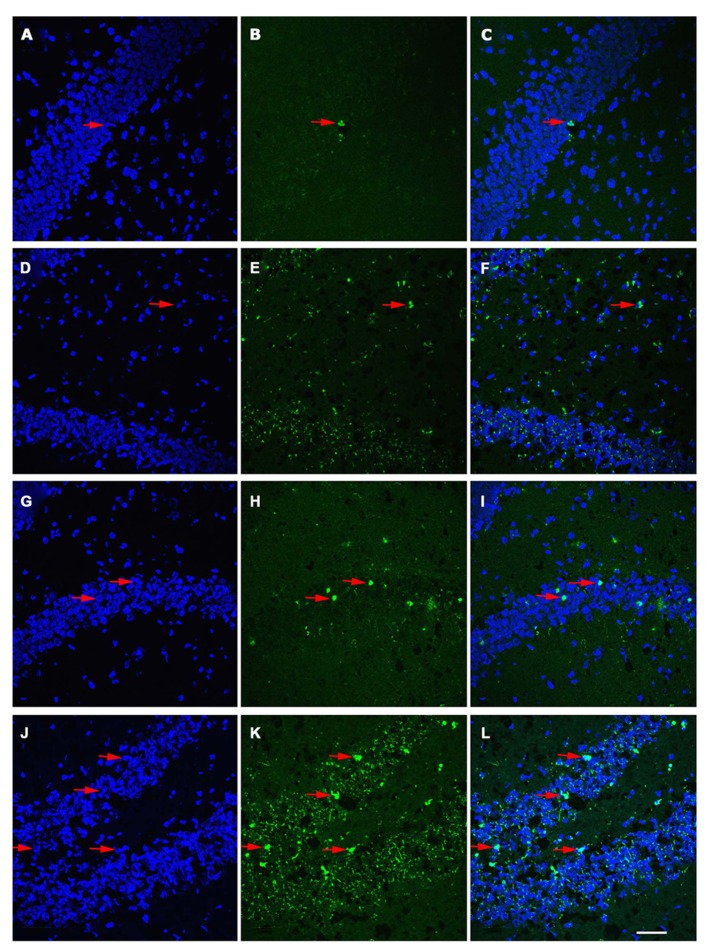
**Distribution of TUNEL-labeled apoptotic cells in the dentate gyrus (DG) of juvenile and aged rats following TBI.** Confocal images showing TUNEL-labeled (green) apoptotic cells counterstained with DAPI (blue) in the hippocampus of a juvenile sham **(A–C)**, aged sham **(D–F)**, juvenile injured **(G–I)**, and an aged injured rat **(J–L)** at 48 h following injury. DAPI-stained sections **(A,D,G,J)** label the nuclei within the DG, while TUNEL-labeling **(B,E,H,K)** reveals apoptotic cells in this region. Co-localization of TUNEL/ DAPI **(C,F,I,L) **in conjunction with morphological analysis provides verification of their apoptotic phenotype. It should be noted that sections from aged brain in both sham and injured aged animals **(E,F,K,L)** have high levels of background staining as a result of the autoflourescence of lipofuscin, pigment granule product found in neurons that is associated with aging. Arrows denote apoptotic cells. Scale bar = 50 μm.

**FIGURE 3:**
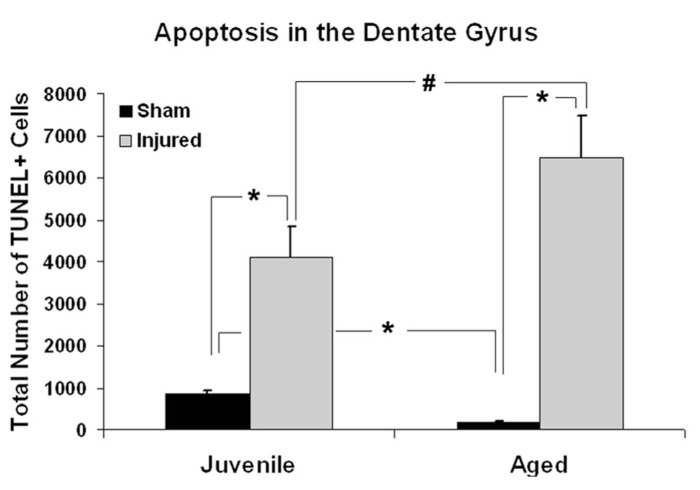
**Quantitative analysis of the apoptotic response in the dentate gyrus (DG) following aging and TBI.** Graph showing estimated numbers of TUNEL-labeled cells in the DG of juvenile and aged rats subjected to a FPI or sham injury at 48 h post-injury. In sham animals, juvenile animals displayed approximately five times more TUNEL-positive cells than their aged sham counterparts (^*^p < 0.01). Following injury, the number of TUNEL-positive apoptotic cells was significantly increased in both juvenile (^*^p < 0.05) and aged animals (^*^p < 0.01) as compare to their age-matched shams. While aged animals exhibited a 38.9-fold increase in the number of TUNEL-labeled cells following TBI, juvenile rats displayed a more modest 4.8-fold increase in apoptotic cell numbers over sham levels in response to injury. The injured aged brain also had significantly higher number of TUNEL-labeled cells than the injured juvenile brain (^#^*p* < 0.05).

Age-associated differences in the apoptotic response of the brain to traumatic insult may represent a mechanistic underpinning of the heightened vulnerability of the aged brain to TBI, as well as contributing to the poor cognitive recovery observed in elderly patients following TBI. In this study, TUNEL labeling was employed to assess the level of apoptosis in the DG of juvenile and aged rats following a moderate lateral fluid percussion or sham injury. Varying levels of TUNEL-positive cells were observed in the DG in all groups assessed (juvenile-sham; aged-sham; juvenile-TBI; aged-TBI), localized predominately in the GCL and subgranular zone (**Figures 1** and **2**). Quantitative analysis to compare the number of TUNEL labeled cells between juvenile and aged animals revealed an age-related difference, both in the uninjured (sham) condition as well as following TBI (**Figure 3**). Specifically, sham juvenile animals exhibited significantly higher levels of apoptosis as compared to aged sham counterparts (**Figure 3**, ^*^p < 0.05). Furthermore, while significantly increased TUNEL labeling was observed in the DG of both age groups following TBI, the magnitude of the injury-induced apoptotic response was significantly more pronounced in aged animals as compared to juveniles (**Figure 3**, *p* < 0.05). By comparing the number of apoptotic cells in the injured brain to the sham base level between age-matched counterparts, the injured-aged brain exhibited a 38.9-fold increase while the injured-juvenile showed a more modest 4.8-fold increase.

### CELLS UNDERGOING APOPTOSIS WERE PREDOMINANTLY NEURONS

**FIGURE 4:**
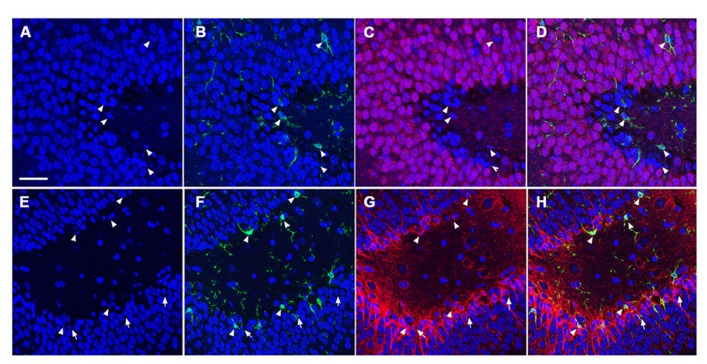
**TUNEL-positive apoptotic cells in the DG of the juvenile rat following TBI are newly generated immature neurons.** Confocal microscopic images show triple labeling of the neuronal marker NeuN (red, **C,D**) or PSA-NCAM (red, **G,H**), the microglia marker Iba1 (green), and the nuclei marker DAPI (blue). **(A–H)** DAPI-labeling revealed apoptotic cell nuclei with condensed, fragmented morphology located mostly in the subgranular zone of the DG (arrows and arrowheads). Many of these nuclei are labeled with Iba1 **(B,D,F,H, **arrowheads**)**, and are NeuN-negative **(C,D)**, but PSA-NCAM-positive **(G,H, **arrows and arrowheads**)** suggesting that the majority of apoptotic cells in the juvenile brain are immature neurons in the subgranular zone of the DG. Scale bar = 30 μm.

**FIGURE 5:**
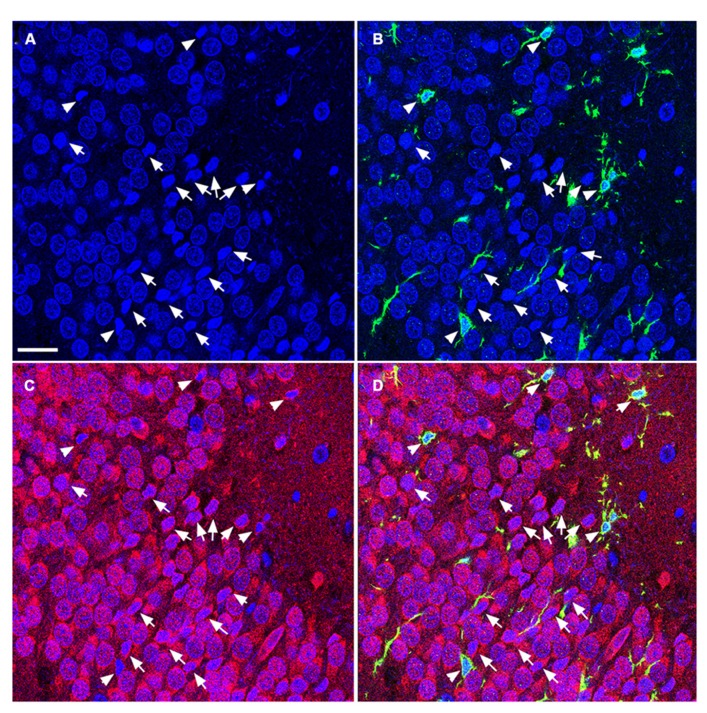
**TUNEL-positive apoptotic cells in the DG of the aged rat following TBI are predominantly mature neurons.** Confocal microscopic images show triple labeling of the neuronal marker NeuN (red), the microglia marker Iba1 (green), and the nuclei marker DAPI (blue). **(A–D)** DAPI-labeling showing cells undergoing apoptosis with condensed, fragmented morphology located in the granule cell layer of the DG (arrows and arrowheads). These cells are NeuN-positive **(C,D**, arrows), some of them are co-labeled with Iba1 **(B,D**, arrowheads) suggesting that the majority of the apoptotic cells in the injured aged brain are mature neurons in close association with microglia, in the granule cell layer of the DG. Scale bar = 60 μm.

To determine the cellular constituents of apoptotic cells in the DG, we used neuronal markers NeuN (for mature neurons) and PSA-NCAM (for immature neurons) and the astrocytic marker GFAP combined with microglial cell marker Iba1. Apoptotic cells were identified by the presence of condensed, fragmented nuclei with DAPI staining (**Figure 1**). The majority of apoptotic cells in the DG of the juvenile rats as characterized by dense DAPI labeling were NeuN-negative, PSA-NCAM-positive immature neurons located in the subgranule layer of the DG (**Figure 4**, arrows). In contrast, there were very few PSA-NCAM-positive cells in the DG in the aged brain. Rather, most of the apoptotic cells with dense DAPI labeling observed in the aged DG were NeuN-positive, PSA-NCAM-negative localized to the GCL (**Figure 5**, arrows). In both age groups, the apoptotic cells were enveloped by Iba+ microglial cells suggesting that the apoptotic cells were taken up by microglial cells (**Figures 4** and **5**, arrows). No GFAP-labeled astrocytes displayed apoptotic nuclei morphology (data not shown).

### THE EXPRESSION LEVEL OF APOPTOSIS-ASSOCIATED PROTEINS IN THE DG CHANGED DURING AGING AND AFTER INJURY

**FIGURE 6:**
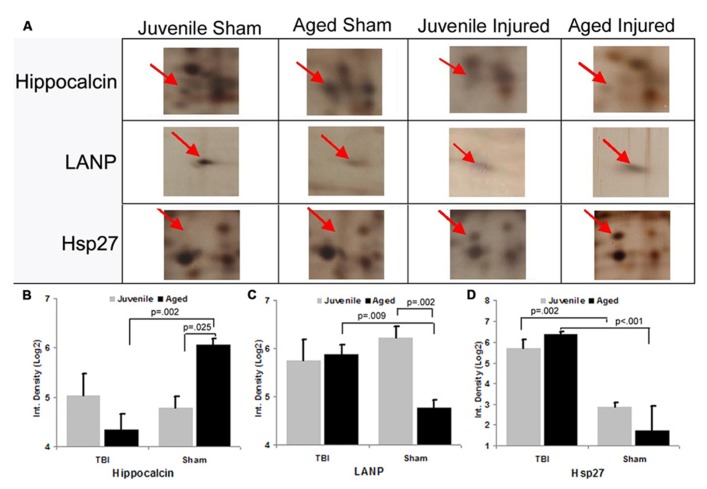
**Differential expression of apoptosis-associated proteins in the dentate gyrus (DG) following aging and injury.** **(A)** Standard format, silver-stained 2D gels showing apoptosis-associated proteins expressed in the DG of juvenile and aged rats in both sham and injured groups at 48 h post-injury. Arrows point to distinct apoptosis-related proteins which exhibit changes in expression as a consequence of aging and/or injury, the identity of which were determined using LC-ESI-MS/MS. **(B–D)** Graph shows the expression levels of apoptosis-related proteins (hippocalcin, LANP and Hsp27) in the DG in relation to normal aging and injury. In sham animals, the expression levels of hippocalcin was increased with aging, while LANP was decreased with aging. HSP27 was low in both age groups. Following TBI, hippocalcin was sharply decreased in the injured aged brain but slightly increased in the juvenile brain. Whereas the level of LANP was increased in the injured aged brain, but slightly decreased in the injured juvenile brain. The expressed level of Hsp27 is drastically increased in both juvenile and aged brain following TBI. Results are reported as the log(2) transformed value of the optical density measure (each unit = doubling in density). Mean ± SE; *n* = 3; ^*^p < 0.05.

To gain insight into the underlying mechanisms of the observed aging/injury-associated differences in the DG apoptotic response, we examined protein expression profiles in the DG in sham and injured juvenile and aged rats. Specifically, each individual protein sample from the DG was subjected to a 2D electrophoresis. Proteins spots from each 2D gel were then semi-quantitatively analyzed to identify apoptosis-associated proteins that are differentially expressed in the DG during aging process as well as following TBI. A comparison of 2D gel protein expression profiles between the experimental groups revealed several age- and injury-induced alterations in the DG proteome (**Figure 6**). Sixty distinct protein spots were detected recurrently across 2D gels with 10 exhibiting a twofold or greater change in abundance with injury or aging as a factor. Using tandem MS, 3 of the 10 gel spots were identified as proteins implicated in apoptotic processes: hippocalcin (P23k), acidic nuclear phosphoprotein pp32 (LANP), and heat shock protein (Hsp27).

**FIGURE 7:**
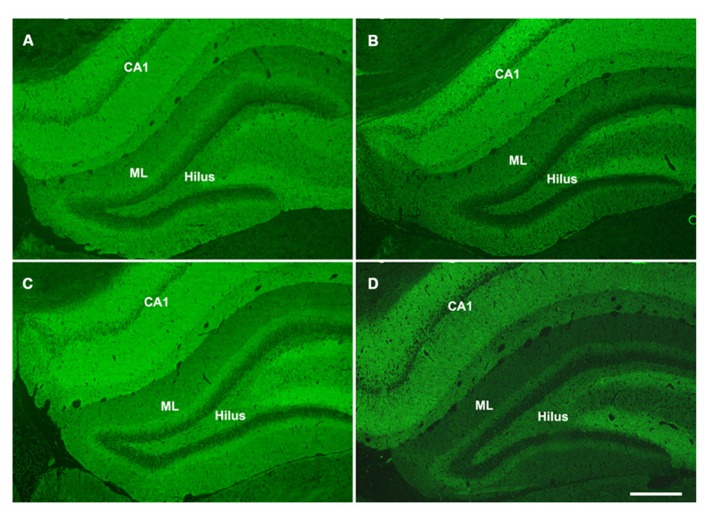
**Hippocalcin expression patterns in the hippocampus of juvenile and aged rats following TBI.** Representative images of hippocalcin immunofluorescent staining of coronal sections of the DG of a juvenile sham **(A)**, aged sham **(C)**, juvenile injured **(B)**, and an aged injured **(D)** rat at 48 h post-injury. Hippocampal CA1 region, molecular layers (ML) and hilus region of the DG show strong hippocalcin staining. The staining was done at the same time for all sections and pictures were taken at the same time with same exposure, minimal adjustment of brightness and contrast was made to maintain the true staining patterns. Note that the intensity of hippocalcin immunoreactivity mirrors the protein expression pattern observed via 2-D gel proteomic analysis. Scale bar = 200 μm.

Hippocalcin, a calcium binding protein that has been shown to protect neurons against apoptosis, exhibited a significantly higher level (2.4-fold; *F* = 6.2; *p* = 0.025) in the DG of sham aged animals as compared to sham juveniles (**Figures 6A,B**). The elevated expression of this anti-apoptotic protein in the aging brain appears related to the declining levels of apoptosis that are observed during the aging process within the DG (as assessed by TUNEL staining). In addition to the differential expression of hippocalcin between sham animals of different ages, alterations in the expression levels of this anti-apoptotic protein were also observed in response to injury. A comparison between sham and injured aged animals revealed a significant decrease (-3.3-fold; *F* = 13.4; *p* = 0.002) in hippocalcin expression following TBI (**Figures 6A,B**) which corresponded to the marked injury-induced increase in TUNEL-positive cells observed in this aged population (**Figure 3**). In contrast to that seen in aged animals, no significant change in hippocalcin expression level was observed in juvenile rats as a consequence of injury. This hippocalcin expression pattern observed by proteomic analysis was further confirmed by immunohistochemistry. As shown in **Figure 7**, strong hippocalcin immunoreactivity was observed in the stratum oriens, stratum lucidum, and stratum radiatum of the CA1 region in all groups but particularly in the sham aged brain (**Figure 7C**). In the DG, hippocalcin was expressed in the molecular layers (ML) and hilus region, with robust expression observed in the aged sham animals (**Figure 7C**) as compared to that of juvenile sham animal (**Figure 7A**). Furthermore, while hippocalcin immunoreactivity remained relatively unchanged in the DG of juvenile animals following injury (**Figure 7B**), there was a substantial decline in hippocalcin immunoreactivity in the DG of aged rats in response to TBI (**Figure 7D**).

LANP, a protein that has been shown to exhibit pro-apoptotic properties, revealed a significantly greater level (2.7-fold; *F* = 14.6; *p* = 0.002) in the DG of juvenile sham animals as compared to aged shams (**Figures 6A,C**). The significantly decreased expression of this apoptosis-promoting protein in the DG during the normal aging process parallels the declining levels of apoptosis observed with aging (as assessed by TUNEL staining). Furthermore, while no change in LANP abundance was observed in the DG of injured juveniles, a significant increase (2.2-fold; *F* = 9.5; *p* = 0.009) in LANP expression over sham levels was observed in aged animals following TBI (**Figure 6**). This injury-induced increase in LANP expression in the aged DG following TBI corresponds to the markedly enhanced levels of apoptosis in this population.

Unlike the aforementioned proteins, no significant alterations (*F* = 0.2; *p* = 0.638) in Hsp27 expression were observed in the DG as a factor of normal aging (**Figures 6A,D**). However, the expression of this anti-apoptotic/anti-necrotic protein was significantly enhanced (13-fold; *F* = 47; *p* < 0.001) as a factor of injury, irrespective of age.

## DISCUSSION

The aging population is the most vulnerable group to TBI and display heightened levels of cognitive deficits as a result, in part, from the progressive neuronal cell death in the hippocampus. Among the pathological responses that occur following TBI, apoptosis plays an important contributing role to the secondary insults that lead to neuronal loss. In this study, we have observed aging- and injury-related differences in apototosis levels and in the cell types that undergo apoptosis in the DG of the hippocampus. In sham animals, juveniles exhibited higher baseline levels of apoptosis as compared to their aged counterparts. Following injury, the number of apoptotic cells was significantly increased in both age groups with aged animals exhibiting a more marked increase. Furthermore, the cell types that undergo apoptosis, as well as their localization within the DG, were different for the two age groups. In the juvenile brain, the majority of apoptotic cells were newly generated PSA-NCAM+ immature neurons located in the SGZ, whereas in the aged brain, the majority of apoptotic cells were mature neurons residing in the GCL. Using proteomic approaches, we have also identified age-and injury-associated alterations in the expression levels of three apoptotic-related proteins in the DG. Specifically, we observed changes in the expression levels of heat shock protein 27, hippocalcin, and LANP (acidic nuclear phosphoprotein), which suggested the presence of differential regulating pathways of apoptosis in the normal aging process and following TBI.

Apoptosis is an important mechanism during brain development for regulating neuronal cell numbers and to ensure the appropriate formation of neuronal circuitries. Apoptosis also occurs in the neurogenic regions of the adult brain, with a significant portion of newly generated cells in the adult brain eliminated within the first months following generation (Dayer et al., 2003). Cells undergoing apoptosis are removed by resident microglia cells (Sierra et al., 2010). These apoptotic processes that occur in the neurogenic regions of the normal adult brain are thought to be mediated through Bax, Blc pathways. Studies have revealed that transgenic mice with Bax-deficiency or over-expression of bcl-2 show increased numbers of new neurons in the DG, a phenomenon that results from decreased apoptosis and not increased cell proliferation (Sun et al., 2004; Kuhn et al., 2005). The continuous increase of DG cell numbers in Bax-knock out mice resulted in a readjustment of afferent and efferent synaptic connections, with reductions in dendritic arborization, synaptic transmission, and reduced performance in hippocampal-dependent learning and memory functions (Kim et al., 2009). In this context, elimination of excess newly generated neurons via apoptosis in the adult brain is essential for the normal organization and function of the hippocampus. Following aging, the degree of neurogenesis in the DG is sharply decreased as a result of the normal aging process (Rao et al., 2006; Olariu et al., 2007; Walter et al., 2011). Studies have shown that decreased neurogenesis in the DG with aging is accompanied by declines in apoptotic cells in this region (Heine et al., 2004; Sierra et al., 2010). Our current findings in sham animals, which show that TUNEL^+^ cells are predominantly localized to the SGZ and that the number of TUNEL^+^ cells decline with aging, are in agreement with these published studies.

Following TBI, the injured brain undergoes a cascade of secondary events which include apoptosis and neurogenesis. We have previously shown that TBI enhances hippocampal neurogenesis and that this enhancement is more prominent in the juvenile brain as compared to adults (Sun et al., 2005). In the current study, we observed increased TUNEL^+^ cells in the injured juvenile brain as compared to sham counterparts, with apoptotic cells localizing predominantly to the SGZ and displaying an immature neuronal phenotype (PSA-NCAM^+^). This observation is in agreement with previously published findings that adult-generated immature neurons in the SGZ are vulnerable to TBI, with many cells undergoing cell death due to the injury impact (Gao et al., 2008). In the current study, one of the striking findings is the marked increase in TUNEL^+^ cells in the DG of aged animals following TBI. The majority of the dying cells in the injured aged DG area are NeuN^+^ mature neurons localized to the GCL as opposed to the PSA-NCAM^+^ cells in the SGZ, as found in the juvenile DG. These age-related differences reflect changes in the degree of neurogenesis as very few PSA-NCAM^+^ cells are found in the aged brain. The observed cell death of mature neurons in the aged brain is likely due to the vulnerability of the aged brain to TBI. It is known that heightened levels of oxidative stress, inflammation, excitotoxicity, etc. lead to increased neuronal death in the aged brain, particularly in the hippocampus, following TBI (Shao et al., 2006; Sandhir et al., 2008; Timaru-Kast et al., 2012).

Neuronal death following TBI occurs through several processes and via many mechanisms involving both necrosis and apoptosis. Calcium dysregulation, excitotoxicity, activation of cysteine proteases, mitochondrial permeability transition, and mechanical pertubation of neuronal membranes are all mechanisms that contribute to apoptotic and/or necrotic neuronal cell death after TBI (Shapira et al., 1989; Fineman et al., 1993; Zipfel et al., 2000). The precise mechanism that determines the fate of a particular cell type has yet to be precisely defined. The cell type and regional differences in the apoptotic response that we have observed in the aged and juvenile brain following injury suggest that different mechanisms are at play.

Developmental apoptosis is thought to selectively remove unviable cells in order to promote overall growth whereas cell death after TBI may play a much more detrimental role in recovery after injury. One mechanism for modulating apoptosis is a shift between pro and anti-apoptotic factors that promote the expression of proteins responsible for cell death (Raghupathi, 2004). In the current study, using proteomic approaches, we identified aging and injury associated changes of three apoptosis related proteins (Heat shock protein beta-1, Hippocalcin, LANP) which may be responsible for the differences observed in the aging and injury-induced apoptotic response.

Heat shock protein beta-1 (Hsp27) was present on 2D-electrophoresis gels run for this study at a MW of 23 kDa and with an isoelectric point (pI) of 6.12. In our study, Hsp27 expression level was low in sham animals regardless of age. Its expression level was drastically increased after injury in both age groups indicating that Hsp27 is an injury-induced protein, which is in agreement with previous findings that report enhanced synthesis of Hsp27 after stress. To date, no published literature has reported HsP27 expression following TBI. Hsp27 is a member of the small heat shock or stress protein (shsp) families that are known to display enhanced synthesis after heat or oxidative stress (Landry et al., 1989; Mehlen et al., 1995). The increased production of hsp27 following TBI in both juvenile and aged brain may represent a protective response of the CNS following injury-induced excitotoxicity, oxidative stress, and inflammation. Hsp27 functions as molecular chaperones (Horwitz, 1992) or actin capping/decapping enzymes (Guay et al., 1997) involved in several fundamental cellular processes including protein intracellular transport, cytoskeleton architecture, translation regulation, intracellular redox homeostasis, and most relevant to the current study, protection against spontaneous or stimulated programmed cell death (reviewed by Vidyasagar et al., 2012). Hsp27 has been shown to have significant anti-apoptotic properties via different pathways including Fas-FasL, Bax, and cytochrome *c*, as well as caspase-dependent apoptosis (Bruey et al., 2000; Charette and Landry, 2000; Havasi et al., 2008).

Hsp27 has also been studied in relation to necrosis, a cell death process that has been shown to dominate after moderate to severe experimental TBI (Conti et al., 1998). Necrosis has been shown to occur more often in the CA1, CA3, and hilus regions of the hippocampus after TBI, whereas apoptosis has been shown to occur more often in the DG (Clark et al., 1997). Over expression of hsp27 protects against both programmed cell death and necrosis (Wagstaff et al., 1999). Hsp27 expression has been shown to decrease intracellular reactive oxygen species levels, a condition that often triggers necrosis. Hsp27 has also been shown to block cell death induced by TNFα by increasing cellular content of the antioxidant glutathione (Mehlen et al., 1995, 1996). In addition, shsp expression has been shown to protect against cellular necrosis induced by oxidative stress (Mehlen et al., 1993), and inflammatory cytokines (Mehlen et al., 1996). The increased production of Hsp27 following TBI in both juvenile and aged brain observed in our study suggests that the brain exerts a protective effect against excessive cell death induced by excitotoxicity, oxidative stress, and inflammation following injury. Consequently, an increased exogenous expression of hsp27 may be a potential therapeutic target for the prevention of massive cell death after neurotrauma specifically via protection against apoptosis, necrosis, and neurodegeneration.

Hippocalcin, also known as p23k, was present on 2D-electrophoresis gels at a MW of 23 kDa and with a pI of 4.87. This protein displayed an increase in expression with aging in sham animals, and a decrease in expression after injury in the aged brain only. The differential expression correlating with aging is in conflict with a previously published study reporting decreased hippocalcin immunostaining in the hippocampus of aged rats during normal aging (Furuta et al., 1999). This discrepancy may due to the differences of sub-regional expression of hippocalcin and the method used. To date no other published study has reported the changes of hippocalcin expression level following injury in aged animals.

Hippocalcin is a member of the small neuronal calcium-sensor family (NCS) (Kobayashi et al., 2005; Palmer et al., 2005). In the hippocampus, hippocalcin is strongly expressed in the pyramidal cell layer and is modest in the DG (Furuta et al., 1999). Although the physiological role of hippocalcin is not completely understood, it is implicated in regulating neuronal viability and plasticity. For example, studies have found that hippocalcin can protect hippocampal neurons against excitotoxicity induced damage by enhancing Ca^+^^+^ extrusion and maintaining ideal intracellular Ca^+^^+^ levels (Masuo et al., 2007). It is also reported that hippocalcin acts to abate apoptosis by interfering with the programmed cell death cascades. For example, hippocalcin interacts with neuronal apoptosis inhibitory protein to protect neurons against Ca^+^^+^-induced cells death by decreasing caspase 3 and caspase 7 activities (Mercer et al., 2000). In addition, hippocalcin has been shown to protect against caspase 12-induced and age-dependent neuronal degeneration (Korkohonene et al., 2005). It also plays a critical Ca^+^^+^-sensing role in NMDA receptor-mediated hippocampal LTD (Palmer et al., 2005) suggesting that hippocalcin may be involved in the downstream Ca2^+^^+^-signaling cascade leading to synaptic plasticity and learning and memory function.

In the current study, in the injured brain, hippocalcin level was sharply decreased in the aged DG while remaining constant in the juvenile animals. This decrease of hippocalcin expression in the injured aged DG corresponds to the sharp increase in the number of TUNEL^+^ cells observed. As hippocalcin plays roles in neuronal viability and plasticity, the decreased expression of hippocalcin in the DG following injury may contribute to the vulnerability of the aged brain to TBI leading to increased neuronal cell death and cognitive dysfunction. Taken together, our findings suggest that hippocalcin may play a role in endogenous repair or homeostasis after TBI.

LANP, also known as ANP21-A, PHAPI (putative HLA-associated protein 1), and pp32 (phosphoprotein with a molecular mass of 32 kDa), was present on 2D-electrophoresis at a MW of 29 kDa and with a pI of 4.00. In sham animals, this protein was expressed at high level in the DG of juvenile brain and was low in the aged brain. Following TBI, an increase in expression was observed only in the aged animal while remaining constant in the juvenile animal. This expression pattern suggests that the aged brain was stimulated to produce high level of LANP to recapitulate the level in the juvenile brain.

LANP is a nucleocytoplasmic shuttling protein with a diverse functions including signaling, protein degradation, cytoskeletal dynamics, and morphogenesis, due to the leucine rich repeat domains which serve as versatile protein binding sites (Kobe and Deisenhofer, 1994). Functionally, the most defined biological function of LANP is its role as a tumor suppressor owning to its apoptotic enhancer function by stimulating apoptosome-mediated caspase activation (Pan et al., 2009). Other known functions of LANP include inhibition of protein phosphatase 2A (PP2A) and histone acetyltransferase (HAT) (Habrukowich et al., 2010). In the brain, LANP is abundantly expressed during early weeks of postnatal life and the level is decreased in adult (Matsuoka et al., 1994). To date there is no published study reporting the changes of LANP expression in the DG related to aging and following brain injury. Consequently, its role in the injured brain is unknown. In the current study, in sham animals, we found a high level of expression of LANP in the DG of juvenile brain and low in the aged. This expression pattern is in agreement with published report about its expression in the cerebella (Matsuoka et al., 1994). In the injured brain, we found a sharply increased expression of LANP in the aged brain following injury. The high expression level of LANP in the sham juvenile and injured aged DG is in parallel with the higher level of TUNEL^+^ cells observed in these groups, which support the role of LANP as an apoptosis promoter. Nevertheless, other roles of LANP such as its function in regulating neurite extension (Kular et al., 2009), COX-2 (cyclooxygenase-2) by interaction with sphingoshine (Habrukowich et al., 2010) may be also at play.

In summary, this study revealed the degree of apoptosis and the differential changes of three apoptosis-related proteins in the DG during normal aging and following TBI. The present design focused on proteins with a sizeable twofold or greater change in level as a response to injury or aging, precluding the detection of more subtle changes in the proteome. Also, the relative lower animal number (three per group), we may miss out on some other proteins that may have significant association to age and injury related changes. Nevertheless, the differential expression of the three apoptosis proteins that we have identified in the current study may suggest any one of these proteins may play an important role in contributing to the decreased capacity of recovery of the aged population following injury. Our results suggest that therapeutic strategies for treating TBI sufferers need to consider age-related differences in pathological changes and cellular pathways in order to be effective for the particular age group.

## Conflict of Interest Statement

The authors declare that the research was conducted in the absence of any commercial or financial relationships that could be construed as a potential conflict of interest.
